# Comparing summary measures of quality of care for family planning in Haiti, Malawi, and Tanzania

**DOI:** 10.1371/journal.pone.0217547

**Published:** 2019-06-07

**Authors:** Lindsay Mallick, Gheda Temsah, Wenjuan Wang

**Affiliations:** 1 The Demographic and Health Surveys (DHS) Program, Avenir Health, Rockville, MD, United States of America; 2 Program Assistance, Design, Monitoring and Evaluation for Sustainable Development (PADMES), ICF, Washington, DC, United States of America; 3 The DHS Program, ICF, Rockville, MD, United States of America; TNO, NETHERLANDS

## Abstract

Measuring quality of care in family planning services is essential for policymakers and stakeholders. However, there is limited agreement on which mathematical approaches are best able to summarize quality of care. Our study used data from recent Service Provision Assessment surveys in Haiti, Malawi, and Tanzania to compare three methods commonly used to create summary indices of quality of care—a simple additive, a weighted additive that applies equal weights among domains, and principal components analysis (PCA) based methods. The PCA results indicated that the first component cannot sufficiently summarize quality of care. For each scoring method, we categorized family planning facilities into low, medium, and high quality and assessed the agreement with Cohen’s kappa coefficient between pairs of scores. We found that the agreement was generally highest between the simple additive and PCA rankings. Given the limitations of simple additive measures, and the findings of the PCA, we suggest using a weighted additive method.

## Introduction

Experts theorize that high-quality care for family planning has the potential to influence reproductive and fertility intentions [1, 2] and can consequently affect contraceptive use [3, 4]. Research in the last several decades has focused on both defining the critical elements of quality of care and assessing the impact of those elements [5–7].

The formative research of Donabedian [8] conceptualized the construct of quality of care, which identifies three core domains: *structure*, or the physical attributes, resources, or infrastructure of a facility, *process* or how the provider interacts with clients during visits, and *outcomes*, which refer to result of the visit, including contraceptive uptake, continuation, or client satisfaction [8, 9]. In 1990, Bruce and Jain identified six elements of family planning services that are critical to quality of care from the client’s perspective: method choice, information given to contraceptive adopters and users, provider competence, interpersonal relations, follow-up planning, and access to related services [6]. Since then, research has continued to better define and adapt these elements over time [4, 10].

Given the convenience of one single measurement, condensing these elements into one summary measure or index is commonly practiced when measuring quality of care for family planning, especially when attempting to associate quality of care with behavioral outcomes such as contraceptive use [11]. Summary scores simplify complex data to a smaller set of variables for comparison purposes or for benchmarking performance within facilities and over time [12–15]. Any tool used to collect information on the quality of care should allow researchers to construct easily understandable output that can help ascertain levels of quality and ultimately lead to quality improvements [16]. Yet research differs in both selection of indicators and their methods for summarizing indicators into aggregate measures [1, 2, 13, 17–20].

Some studies take a simple additive approach to create an overall score by summing or averaging a set of dichotomous variables. For example, one study used data from the 2013–14 Malawi Service Provision Assessment (SPA) to form two indices of quality using this approach [19]. The first index summarized provision of care by summing the “yes” responses referring to specific procedures completed as observed during the visit. The second index drew from client responses in an exit interview, assigned values to categorical responses, and then summed these values to assess experience of care [19]. In another study, a health facility-based panel study in the Philippines, researchers identified a positive association between high quality of care and contraceptive continuation by using a simple additive measure of quality [2].

Additive indices are generally easy to construct. These measures foster an intuitive understanding to the concept of quality: with each additional component of quality within a facility, the higher the quality. However, the simple additive approach assumes that each indicator holds equal weight in the concept of quality; correlations and redundancies between variables may bias scores. These scales can create non-normal distributions, with heapings around groups of scores, which can be problematic when categorizing scores into quantiles [13]. Further, the method assumes the construct itself is unidimensional. Thus a simple additive summary score is not always conceptually meaningful and may not accurately portray overall quality of care [21].

A mathematically simple solution to address the issues that arise with the use of a simple additive summary score (unidimensionality and collinearity) is the weighted additive measure. This technique, which reduces the relative importance of variables within a domain while equally weighting domains, is also easy to calculate and interpret. The weighted additive scoring mechanism is based on a pre-determined, dimensional conceptualization. Using health facility data from Peru, Mensch, Arends-Kuenning, and Jain [1] created two weighted additive indices of quality of care based on the Bruce-Jain framework. The first weighted additive index comprised 150 structure-related variables and the second used both structure and process indicators. Variables within domains were summarized and domain scores were added to create an overall score [1]. Others since have applied weighted additive indices of quality of care for family planning [3, 18, 20, 22, 23]. Nonetheless, since a weighted additive index has not been validated, there are still remaining assumptions that the dimensions do in fact carry equal weights.

A third method to summarizing quality of care indicators is principal components analysis (PCA). PCA is a data reduction method used to obtain a smaller set of uncorrelated variables from a large number of correlated variables while maintaining maximum variation from the original set [24]. Researchers can use PCA to understand the underlying structure and critical dimensions of the data. According to the Kaiser criterion, all components with an eigenvalue—a measure of variance explained by the component—of one or higher are deemed important to the underlying construct [25]. The greater the number of components needed to reach an eigenvalue of one, the greater the dimensionality of a construct.

In PCA, the loadings of variables for each component represent the importance of the specific item to the respective component. When calculating an index using PCA, the loadings of the variables on the first component serve as weights for each variable; each item is multiplied by its loading and the weighted items are summed to produce a score. Unlike the weighted additive scoring measures, the weights assigned with PCA reflect the underlying variation of the data and are not preemptively determined. The first component is used as it explains the most variation in a construct; therefore, how strongly the principal component corresponds to the latent construct, how much variance of the latent construct is captured by the principal component, and how dimensional the overall construct is are critical considerations when using PCA to create an index.

In a critique of using PCA to create asset-based wealth indices [26], a PCA-based index resulted in a misclassification of over 50% of households into the wrong wealth quintile when the first component explained less than 30% of the total variance. This suggests that the first component alone may not represent the overall construct or sufficiently capture the variability or dimensionality of household wealth and may result in misrepresentation of the latent construct in question [26]. Yet not all research reports these statistics when creating PCA-based indices.

Bellows et al. [13] used PCA to develop indices with 35 indicators of service readiness for family planning, reflecting the structural aspect of quality, using data from the 2010 Kenya SPA. The authors applied these PCA-based item loadings as weights for indicators assessed in a prior survey in Kenya and compared the two [13]. PCA was also used to create a composite quality index based on a number of structural and process indicators in a study that assessed the association between contraceptive use and quality of family planning services in public facilities [27]. The authors found that the first component resulted from PCA explained a large proportion of variance (63–82%) across the four study countries. Although Bellows et al (2016) did not report the proportion of variation explained by the first component, it was the only study of note that has assessed the sensitivity of a service readiness index that used PCA by comparing it with a simple additive score, finding strong correlation between the PCA-based index and the summative index.

To the authors’ knowledge, no studies have conducted a comparison of all these methodologies of summarizing quality of care of family planning although some research has explored the advantages and disadvantages to creating composite measures of provider performance and how different methods result with different profiling of health care performance [15]. Our study used SPA data from Haiti, Malawi, and Tanzania to compare additive scoring methods with PCA. We compared these different scoring mechanisms in the ease of their construction, the appropriateness of their application, and the comparability in their classification of facilities into different levels of quality categories.

## Data

Our study examines data from health facilities assessed by SPA surveys carried out in Haiti in 2013, Malawi in 2013–14, and Tanzania in 2014–15. These three countries had well timed SPA and Demographic and Health Surveys (DHS) surveys that allowed for ecological linkage between the surveys in each country for the technical report from which this manuscript is based [28]. The SPA surveys comprise 4 separate questionnaires that assess service availability and readiness of facilities, health worker demographics and training, observation of selected client visits, and an exit interview assessing client demographics and perception of the visit.

The SPA survey collects data through interviewing nationally representative samples of facilities, health workers, and clients in a country. In some countries, such as Malawi and Haiti, the SPA is a census of all formal sector health facilities. More details on the sample design of each specific SPA can be found in the surveys’ final reports [29–31]. This report focuses on facilities that provide family planning services, health workers providing family planning care, and clients attending the facility for family planning and who left the facility with a contraceptive method on the day of the survey. After applying respective facility, client, and provider weights, in Haiti, there were 405 facilities with family planning services and clients observed, 1,069 health workers, and 1,212 female clients. In Haiti, there were an average of 3 clients observed at each facility, with a range from 1 to 15 clients. For Malawi, the analysis covered 371 facilities, 865 health workers, and 1,482 women; an average of 4 clients were observed per facility (range 1 to 54). For Tanzania, we analyzed a total of 398 facilities, 1,939 health workers, and 1,686 women; each facility had an average of 3 clients observed at each facility, ranging from 1 to 27 clients.

## Methods

This study uses secondary data originally collected through The Service Provision Assessment (SPA) survey by The Demographic and Health Surveys (DHS) Program. The SPA survey protocols were reviewed and approved by the ICF Review Board and the Ethics Review Committee of respective countries included in this study (Haiti, Malawi, and Tanzania). Data include no personally identifiable information. The methods described here are described in more detail in the corresponding technical report [28].

### Indicators

Previous studies of quality of care for family planning informed our choice of domains and indicators. We assimilated the domains used in existing literature [4, 6, 10]. We structured the indices into eight domains. Table 1 shows a summary of these family planning-specific domains and indicators as well as how they align with Donabedian’s framework; Table 1 also specifies the instrument or source of data for each indicator.

**Table 1:** Summary of domains and indicators used to create indices of quality of care [28].

Domain	Donabedian component	Number of indicators	SPA questionnaire (source of data)	Indicator summary
Choice of methods	Structure	1	Facility	Availability of a mix of methods
	Process	2	Observation	Provider discusses methods/choice
Constellation of services	Structure	5	Facility	Availability of other services: ANC, PNC, HIV, PMTCT, STI
Management	Structure	6	Facility	Supervision and human resource management, contraceptive commodity management
Infrastructure	Structure	17	Facility	General infrastructure (water, electricity, toilet, etc.) and family planning infrastructure (exam room and supplies for family-planning related procedures, including sanitation)
Provider/ technical competence	Structure	1	Health worker	Provider training
	Process	12	Observation	Assessment of reproductive history, fertility intentions, and physical health; review of client card
Follow-up	Process	1	Observation and exit interview	Provider gives information on when to return
Information given to client	Process	2	Observation and exit interview	Provider gives information on method use and side-effects
Process	Client-provider relations	4	Observation and exit interview	Treatment of the client during the visit

We structured the 53 indicators into eight domains summarized in Table 1. We defined the structure-related indicators such that facilities must have certain structure or supply, and it was functioning (or not expired) on the day of the survey [28]. Availability of a mix of methods refers to whether a facility has at least one short acting (pill or injectable), one long-acting reversible or permanent method (implant, IUD, or sterilization), and one barrier method (condom) available. For variables reflecting the constellation of services domain, we considered co-location of services a measure of structural integration. Although co-location of services does not guarantee that a provider will integrate services during the visit, services co-located in one facility is a critical component of integration [32]. Quality assurance measures refer to whether the facility routinely conducts and has documentation of report or minutes from a quality assurance meeting, a supervisory checklist, a mortality review, or an audit of records or registers.

Several process indicators that assess the client-provider interaction during the visit were recorded by the interviewer in the observation of the visit and also assessed via self-report in the client exit interview [28]; prior research recommends combining responses to construct these indicators [5]. Neither observation of visits nor exit interviews alone may reflect adequate provider counseling during a visit; clients may not fully retain or comprehend the information provided, signifying that the providers did not counsel effectively [33]. Therefore, we constructed three process indicators contingent upon concordance between exit interview responses and interviewer’s observation. For example, we positively coded counseling on side effects only if both the interviewer noted observed counseling and the client reported receiving counseling. The other two indicators include: provider informed client when to return and provider explains how to use the selected method. [28]

While *outcome* is an important domain of quality of care, we did not include outcome measures in this study for a number of reasons. First, SPA surveys lack robust outcome measures. While client satisfaction is assessed, this measure is highly biased [34]. Additionally, we omitted outcome measures from this index to allow for future exploration of the association between these indices and alternate outcome measures from DHS surveys [28].

We created the indices at the facility level among facilities that had observations of family planning clients on the day of the survey [28]. We collapsed provider or client data around the mean for each facility, merged into the facility file, then dichotomized facilities as either at and above or below the mean for all facilities for that indicator [4, 28]. Given that most facilities had only an average of 3–4 clients observed, the indicators were prone to skewness. All items included in the indices were binary. Cases with missing information were coded as 0 under the assumption they did not exist or occur.

### Index creation

The simple additive index is a sum of all the items with a potential range between 0 and 53 [28]. For the weighted additive index, we first calculated the domain scores by adding the indicators within each domain; then we divided the sum in each domain by the number of indicators in that domain, multiplied by 100, and divided by the total number of domains [28]. To create the total weighted additive score, we summed the eight domain scores for a possible score ranging between 0 to 100.

Unless otherwise specified, PCA formulates principal components that are not correlated with each other. In the multidimensional space on which PCA operates, this calculation entails that the axes are perpendicularly (orthogonally) situated in relation to each other. Given that the dimensions of quality of care are likely correlated, we tested an oblique rotation of the axes calculated by the PCA, which allows the axes to lie at any angle in relation to other axes in the multidimensional space. We ultimately applied an unrotated, unweighted PCA, using the Stata command *pca* as the loadings were most consistent with theory without rotation. We created an overall score for each facility with the loadings from the first component [28]. We did not impose a loading threshold for the inclusion of items as the purpose of our study was not to reduce the number of indicators.

### Categorizing scores

Using tercile cut points, we categorized the continuous scores from the three different approaches as low, medium, or high in order to understand more intuitively how facilities are ranked according to each score. Social scientists commonly dichotomize continuous variables as a way to simplify their analysis and interpretation of results, although statisticians often criticize this practice. Dichotomization results in a loss of statistical power and thus the ability to detect significant differences [35, 36]. When applying a median split, values below the median are treated as equal and equally dissimilar from the values above the median. Alternatively, analysts can categorize continuous variables into terciles, quartiles, or quintiles. This allows for comparison between the lowest and the highest groups and results in less efficacy lost than dichotomizing a normally distributed continuous variable [37]. While categorizing scores into more than three quantiles is feasible, and such an approach adopted has been adopted for other well-known indices such as the DHS Wealth Index, fewer categories may be preferred from a programmatic or decision-making standpoint [38].

### Comparison of scores

As described in Mallick, Wang, and Temsah [28], we calculated the percent agreement and Cohen’s kappa coefficient between pairs of scores by using the *kappa* command in Stata 14. Cohen’s kappa adjusts for agreement due to chance is a statistic that ranges between -1.0 and 1.0. The greater the value, the higher the agreement; discrete categories can describe levels of agreement. Near perfect agreement is achieved when the estimate falls between 0.81–1.0, good agreement falls between 0.61–0.80, moderate agreement is within the range of 0.41–.60, fair agreement occurs between 0.21–0.40 and poor is 0.0–0.20. Any estimate that is less than 0.0 indicates the agreement is worse than chance [39]. Finally, we examine the background characteristics among high quality facilities by scoring mechanisms.

All analyses using SPA survey data included weights to account for nonresponse of facilities, health workers, and clients. Sampling weights also account for the complex survey design that ensures the sample of facilities (in Tanzania), clients, and health workers in the survey is nationally representative. All data are publicly available from https://dhsprogram.com.

## Results

**Fig 1:**
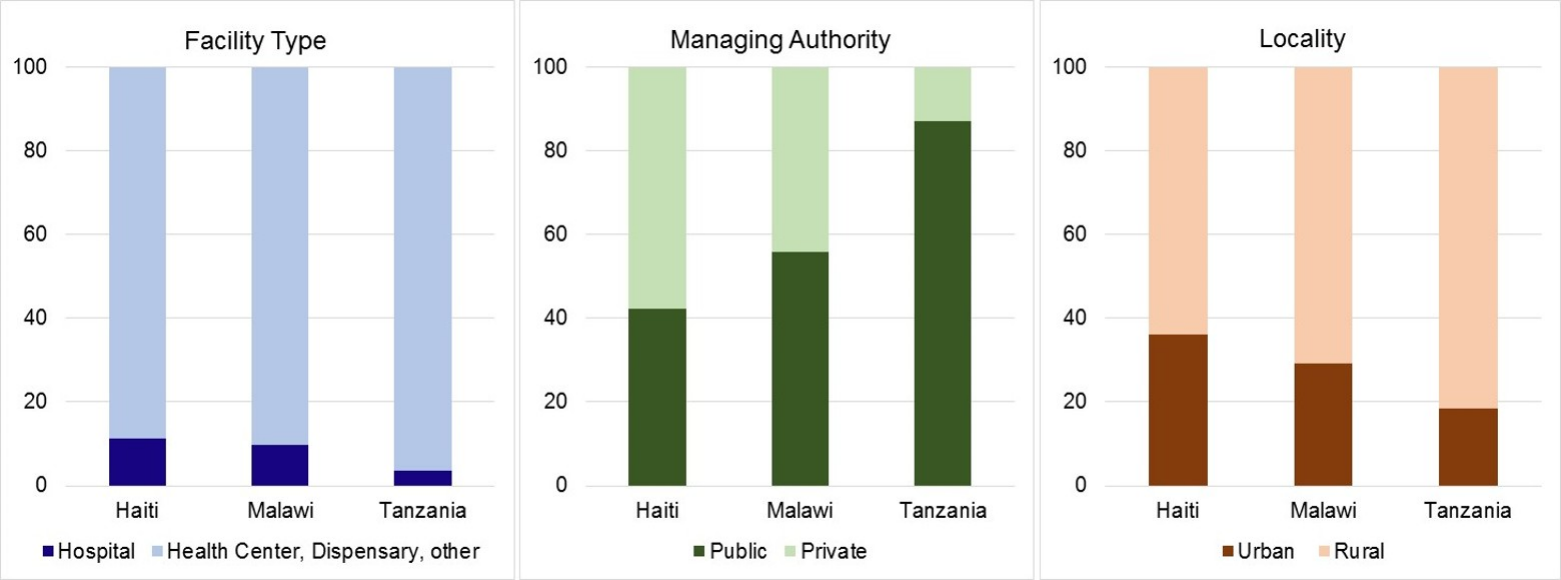
Percent distribution of facilities with family planning services by facility characteristics.

Hospitals constituted less than 15% of all facilities providing family planning services in Haiti, Malawi, and Tanzania (Fig 1). The majority of the facilities were health centers, dispensaries, and clinics. In Haiti and Malawi, the government or private entities managed a similar proportion of facilities. In contrast, in Tanzania nearly 90% of facilities were publicly managed. Facilities in Tanzania were disproportionately located in rural areas, at around 80%. S1 Table shows the distribution of facility characteristics among facilities with family planning clients observed on the day of the survey.

### Indicators of quality of care

#### Structure

Table 2 presents the availability of services, commodities, infrastructure, and management practices related to the structure component of quality of care among facilities providing family planning services with observations of clients on the day of the interview.

**Table 2:** Percentage of facilities providing family planning with structure quality of care items.

	Haiti	Malawi	Tanzania
	% of facilities	% of facilities	% of facilities
Choice of methods			
Mix of methods provided (long acting, short acting, and barrier)	46.3	68.4	60.4
Constellation of services			
With ANC services	98.0	75.3	99.7
With PNC services	90.8	71.1	95.0
With STI services	97.8	99.4	99.2
With HCT services	56.8	90.3	96.5
With PMTCT services	44.9	71.0	95.8
Management			
System for reviewing management/administrative issues	60.8	60.1	72.2
System to obtain client opinions	3.4	9.6	15.1
Supervision in the last 6 months	92.4	84.3	98.6
Inventory of contraceptive supplies	57.5	86.2	66.7
Stock organized by expiration date	4.2	1.6	2.5
Contraceptives protected	66.9	76.5	55.3
Facility infrastructure			
*General*			
Electricity	66.4	61.1	66.0
Water	81.7	95.7	69.7
Toilet	42.0	32.9	38.7
Telephone	23.2	33.9	4.9
Waiting area (protected)	96.3	98.2	93.2
Quality assurance measures in place	7.9	13.5	18.7
*Family planning area*			
Family planning services provided 5 days per week	94.3	71.7	93.0
Private exam room	94.6	97.3	93.2
Digital blood pressure apparatus or cuff and stethoscope	90.1	69.6	79.4
Speculum	3.4	21.1	27.9
Family planning guidelines	61.7	38.4	64.8
Table and stool	69.1	88.0	91.2
Light	16.0	29.2	14.2
Soap	72.3	58.4	66.7
Gloves	58.2	92.0	63.0
Decontamination solution	65.7	58.2	59.9
Sharps box	91.9	92.0	96.4
**Number of facilities**	**405**	**371**	**398**

More facilities that provide family planning services in Malawi (68%) provide a mix of family planning methods with at least one long-acting or permanent method, one short-acting method, and one barrier method than in Haiti (46%) and Tanzania (60%). Family planning facilities with co-located services are common across the three countries; however, fewer facilities in Haiti provide HCT or PMTCT services, likely due to the country’s HIV/AIDS prevalence, which is lower than in Malawi and in Tanzania. Few of these facilities have a system for feedback from clients or have their stock of contraceptives organized by expiration date, ranging from 3 to 15% in each country. The availability of infrastructure-related resources varied, though the most and least common items (telephone, quality assurance measures, a speculum, and a light) were similar across countries.

#### Process

Table 3 presents the quality of care *process* indicators as observed during the visit and as reported by the client as well as one indicator of provider training. This table includes both observation/client and facility-level data for each indicator; although only facility-level indicators were used to create the indices, for ease of interpretation, observation/client-level indicators are discussed here.

**Table 3:** Percentage of clients receiving quality of care items, percentage of providers with training, and percentage of facilities with above average reports of process quality of care items.

	Haiti		Malawi		Tanzania	
	% of clients/ providers	% of facilities above mean	% of clients/ providers	% of facilities above mean	% of clients/ providers	% of facilities above mean
Choice of methods						
Provider mentioned two or more methods	31.8	39.5	32.3	56.6	36.7	60.4
Provider assessed client’s method of choice	33.6	41.7	67.3	45.3	60.9	52.2
Technical/Provider competence						
Client card	91.9	95.4	99.2	95.4	95.1	92.9
*Reproductive history*						
Last delivery date assessed	32.9	41.4	38.9	43.3	51.1	45.3
Pregnancy status assessed	50.1	51.8	34.3	41.6	53.9	45.1
Breastfeeding status assessed	4.4	9.6	17.3	29.6	28.6	38.6
Menstrual cycle regularity assessed	16.7	27.6	21.8	35.0	37.4	41.4
*Fertility intentions*						
Age of client assessed	49.2	51.8	57.7	47.8	76.4	65.9
Current number of children assessed	42.9	45.6	61.4	51.7	79.0	70.3
Desire for more kids assessed	3.3	6.2	23.2	28.2	27.5	31.5
Desired timing for next child assessed	2.6	5.7	15.5	26.4	24.2	31.6
*Physical health*						
Blood pressure measured	78.3	70.5	35.6	41.0	32.5	38.4
Weight measured	53.7	50.5	62.0	54.8	38.0	41.9
Smoking habits assessed	3.7	4.2	1.6	3.7	1.8	4.1
STI symptoms assessed	9.1	17.3	8.3	18.8	11.7	18.3
Chronic illnesses assessed	6.7	12.1	8.4	19.2	24.8	30.2
Follow-up						
Provider told client when to return	75.0	64.0	84.3	66.3	80.6	68.1
Information given to client						
Explains how to use the method	44.7	49.6	50.8	51.6	62.6	60.0
Explains side effects of method	26.6	35.7	38.4	42.0	41.9	45.3
Client-provider relations						
Staff treated client very well	98.0	95.3	92.5	83.2	95.4	90.5
Provider asked if client had questions	52.9	48.1	75.7	65.7	75.4	67.3
Client felt comfortable asking questions	95.5	89.9	90.5	74.7	93.6	90.6
Provider assured client confidentiality	12.7	19.7	21.9	30.4	38.6	43.5
**Number of clients**	**1,212**		**1,482**		**1,686**	
**Number of facilities**		**405**		**371**		**398**
Technical/Provider competence						
Recent training in family planning^1^	34.5	42.4	34.2	45.9	22.0	40.2
**Number of family planning providers**^**1**^	**1,069**		**865**		**1,938**	

^1^All providers for this calculation were not observed giving consultations on the day of the interview

Interviewers observed that providers mentioned two or more methods to only approximately one-third of family planning clients in each of the three countries. In Malawi and Tanzania, close to two-thirds of providers inquired about a client’s preferred method, while only half as many clients in Haiti received this inquiry. Among reproductive history assessment items, inquiring about a client’s pregnancy status was the most common item in Haiti (50%) and Tanzania (54%) while asking about the timing of a client’s last birth was the most common practice in Malawi (39%). Across the three countries, providers infrequently assessed whether or not the client was currently breastfeeding, especially in Haiti where only 4% of women were asked this question. In terms of fertility intentions, provider assessment was also uncommon, particularly regarding asking a woman about her desire for more children and desired timing of next child (only 3% for each in Haiti). Only one third of providers in the three countries have had recent training in family planning.

Interviewers often observed that women were told when to return for follow up; providers were observed to counsel on this for 75% of women in Haiti, 84% in Malawi, and 81% in Tanzania. Only around half of women were told how to use the method (45% of women in Haiti, 51% in Malawi, and 63% in Tanzania) and an even smaller percentage received counseling on side effects (27% in Haiti, 38% in Malawi, and 42% in Tanzania). The most common of all process indicators—staff treated client very well and client felt comfortable asking questions—were nearly universally reported among women in all three countries.

### Results from the Principal Component Analysis

Table 4 shows the results from the PCA, including the loadings, the eigenvalue and the percent of total variance explained by the first component. All items demonstrated low loadings. The highest loading for any item in any country was for the availability of a speculum in Malawi, with a loading of 0.3. In Haiti and Tanzania, the items with the highest loading related to technical competence. In Malawi, a number of variables had negative loadings, most notably those in the constellation of services domain including ANC, PNC, and PMTCT, which suggested an inverse relationship with the latent construct represented by the first component. In fact, these items that had the highest absolute value loaded negatively. In all three countries, the management domain appeared to be one of the least correlated domains with the principal component.

**Table 4:** Loadings of the first component of a PCA, eigenvalue, and % of variance explained.

*Domain*	Indicator	Haiti	Malawi	Tanzania
*Choice of methods*	Mix of methods provided (one long acting, one short acting, one barrier) and currently available	0.11	0.07	0.10
	Provider mentioned two or more family planning methods	0.20	0.00	0.12
	Provider asked about client’s method of choice	0.19	0.07	0.14
*Constellation of services*	With ANC services	0.06	-0.30	-0.05
	With PNC services	-0.01	-0.29	0.00
	With STI services	0.07	0.00	-0.04
	With HCT services	0.19	-0.10	0.04
	With PMTCT services	0.18	-0.28	-0.03
*Management*	System for reviewing management/administrative issues	-0.03	-0.14	0.04
	System to obtain client opinions	0.06	0.01	0.12
	Supervision in the last 6 months	0.04	-0.03	0.04
	Inventory of contraceptive supplies	0.05	-0.07	0.05
	Stock organized by expiration date	0.02	0.04	0.01
	Contraceptives protected from water, sun, pests	0.01	0.06	0.01
*Infrastructure* *(general)*	Electricity	0.12	-0.05	0.07
	Water	0.05	0.05	0.06
	Toilet	0.12	0.19	0.08
	Telephone	0.20	0.23	0.14
	Waiting area (protected)	0.03	0.00	0.05
	Quality assurance measures in place	0.09	0.09	0.19
*Infrastructure* *(family planning area)*	Family planning services provided 5 days per week	0.00	0.17	0.05
	Private exam room	0.00	0.04	0.07
	Digital blood pressure apparatus or a cuff and stethoscope	0.02	0.15	0.03
	Speculum	0.11	0.30	0.21
	Family planning guidelines	0.05	0.07	0.09
	Table and stool	-0.03	0.06	0.04
	Light	0.01	0.23	0.14
	Soap	-0.02	0.23	0.13
	Gloves	0.06	0.10	0.16
	Decontamination solution	-0.03	0.17	0.11
	Sharps box	0.01	-0.02	0.04
*Technical/Provider competence (general)*	Client card	0.07	-0.12	0.05
	Recent training in family planning provision	0.07	0.08	0.04
*Technical competence (reproductive history)*	Last delivery date assessed	0.27	0.13	0.27
	Pregnancy status assessed	0.21	0.13	0.26
	Breastfeeding status assessed	0.17	0.09	0.23
	Menstrual cycle regularity assessed	0.21	0.19	0.24
*Technical competence (fertility intentions)*	Age of client assessed	0.23	0.13	0.15
	Current number of children assessed	0.25	0.16	0.13
	Desire for more kids assessed	0.19	0.16	0.26
	Desired timing for next child assessed	0.18	0.13	0.22
*Technical competence (physical health)*	Blood pressure measured	0.12	0.17	0.21
	Weight measured	0.14	0.03	0.19
	Smoking habits assessed	0.20	0.08	0.14
	STI symptoms assessed	0.22	0.12	0.17
	Chronic illnesses assessed	0.25	0.13	0.21
*Follow-up*	Provider informed client when to return	0.07	0.02	0.09
*Information given to client*	Explains how to use the selected method	0.10	0.02	0.16
	Explains side effects of selected method	0.26	0.05	0.22
*Client-provider relations*	Staff treated client very well	-0.06	0.09	-0.01
	Provider asked if client had any questions or concerns	0.14	0.09	0.17
	Client felt comfortable asking questions during the visit	-0.13	0.13	0.02
	Provider assured client of confidentiality	0.18	0.07	0.19
**Eigenvalue**		4.22	4.35	5.17
**Percent of total variance**		0.08	0.08	0.10

**Fig 2:**
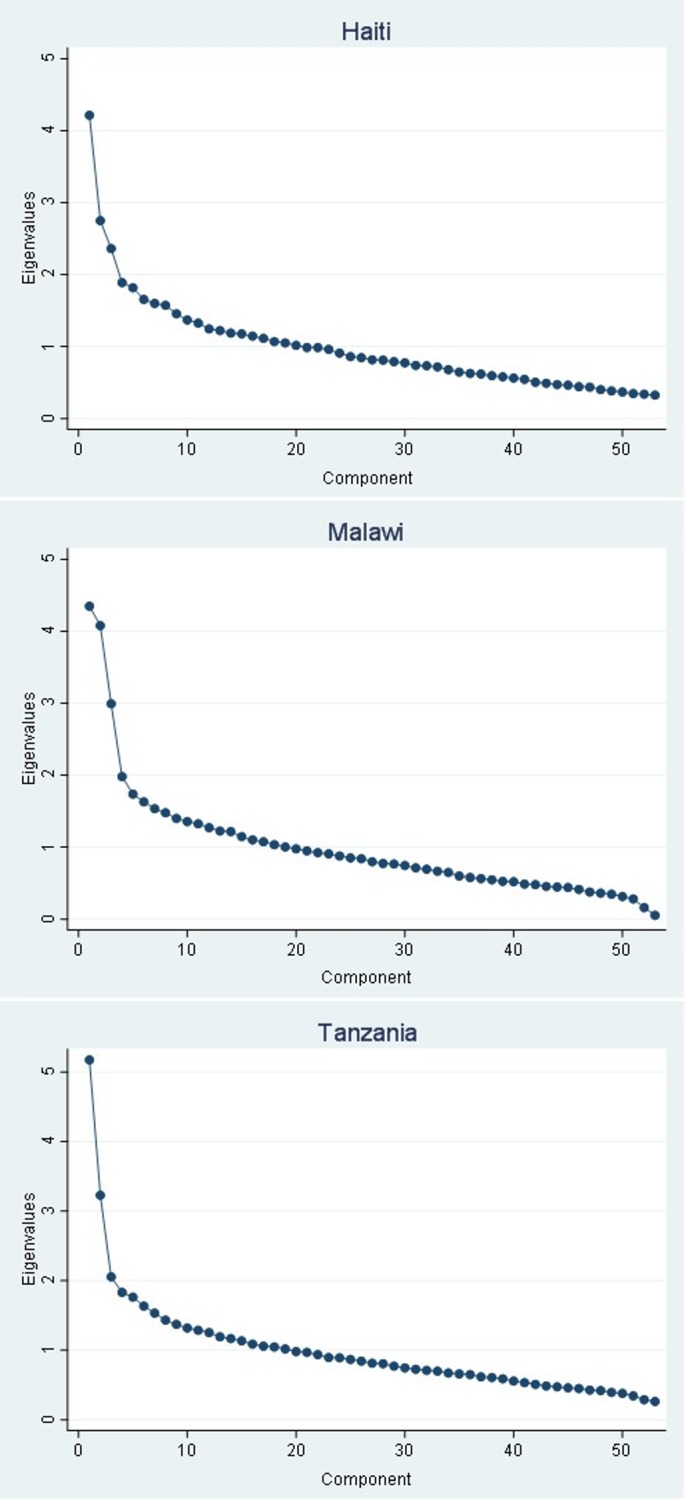
Scree plots of the eigenvalues after PCA.

The percent of variance explained by the first component ranged from 8% to 10%. The low variance, the diverging items with high loadings in each country, and the negative loadings elicit concerns for the use of PCA to create a summary score using the loadings from the first component. Further, in each country, an eigenvalue equal to less than 1 was only reached after the 19^th^ (Malawi, Tanzania) or 20^th^ (Haiti) component, indicating a highly dimensional construct, as seen in the scree plot of the eigenvalues in Fig 2. These scree plots indicate that at least 3 (Tanzania), if not 4 (Haiti, Malawi) components represent important dimensions of the construct.

### Agreement in the categorization of facilities’ quality by scoring mechanism

Table 5 presents the agreement comparisons for the quality of care indices after categorization.

**Table 5:** Percent agreement and Cohen's kappa coefficient among three quality of care scores among health facilities providing family planning services in Haiti, Malawi, and Tanzania.

			Haiti (n = 405)		Malawi (n = 371)		Tanzania (n = 398)	
Index 1	vs	Index 2	% Agreement	Kappa	% Agreement	Kappa	% Agreement	Kappa
Simple additive		Weighted additive	69.6	0.54***	64.0	0.46**	63.8	0.45***
Weighted additive		PCA	57.0	0.36***	39.7	0.10**	64.4	0.45***
PCA		Simple additive	71.1	0.57***	51.2	0.27***	85.3	0.77***

* p < .05

** p < .01

*** p < .001

We found fair to moderate levels of agreement among pairs of scoring mechanisms in Haiti. While the highest (moderate) agreement in Haiti was found in the comparison between the simple additive and PCA score (71% agreement, kappa = 0.57, p<0.001), the simple additive score had moderate agreement with weighted additive score. In Malawi, the simple additive score moderately agreed with the weighted additive score (64% agreement, kappa = 0.46), while there was poor agreement between the weighted additive and PCA scores (40% agreement, kappa = 0.10). In Tanzania, the highest agreement was found between the simple additive score and PCA score, with good agreement (85% agreement, kappa = 0.74, p-value <0.001). In Haiti and Malawi, the lowest agreement occurred between the weighted additive score and the PCA score.

### Differences in facility classifications by facility background characteristics

**Fig 3:**
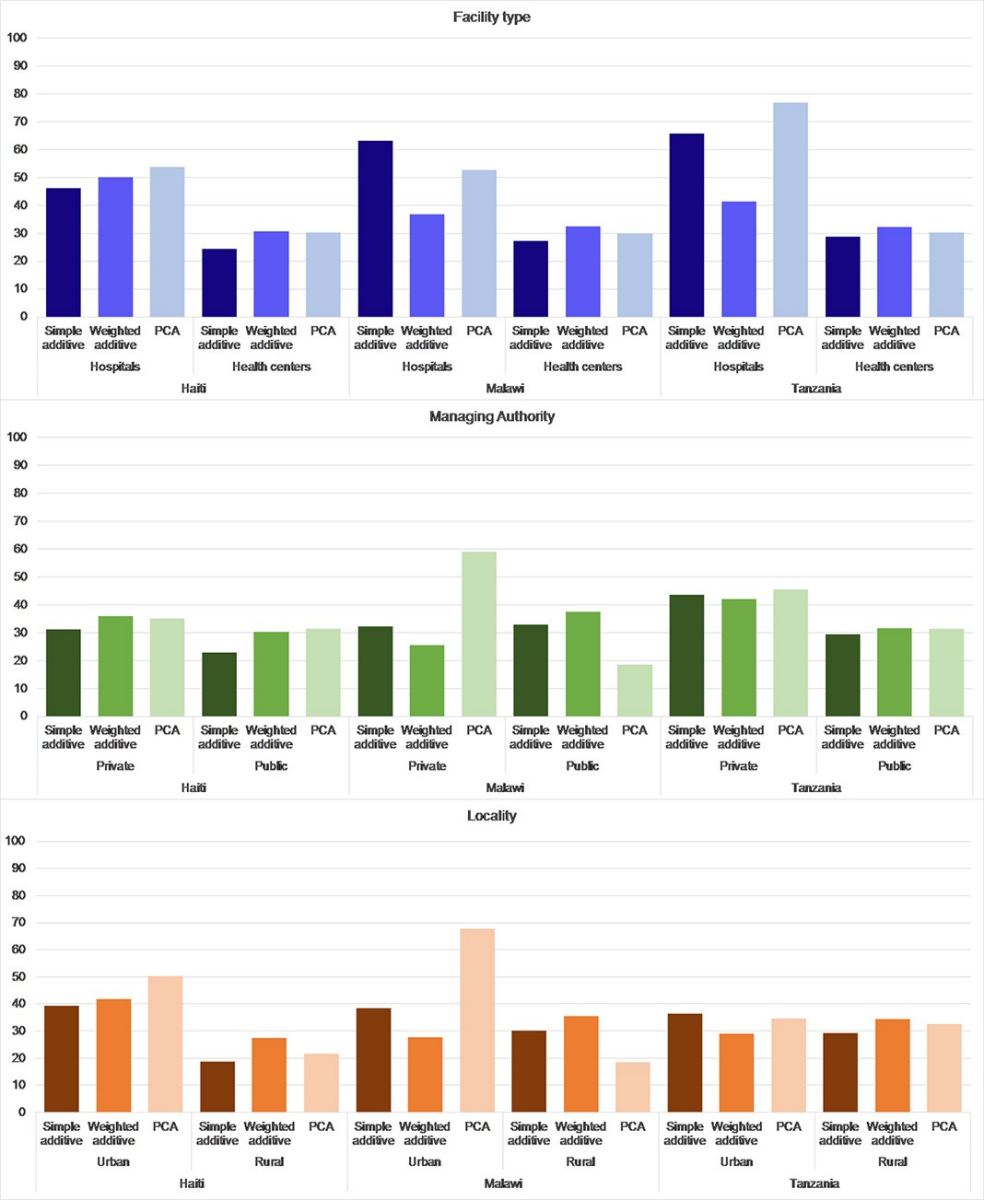
Percent of facilities scored as high quality by scoring mechanism and background characteristic.

The three scoring approaches also result in different facility classifications when stratifying by facility type, managing authority, and locality. Fig 3 (with supporting information provided in S2 Table) shows the distribution of facilities that are ranked as high by each scoring mechanism by their characteristics. We see a large discrepancy in the proportions of hospitals that are ranked as high quality in each country by scoring mechanism. In Malawi, we found that 37% of hospitals are scored high according to the weighted additive approach compared with 63% according to the simple additive approach and 53% according to the PCA approach. A similar discrepancy was found among high scoring hospitals in Tanzania, where the weighted additive scores reduced the proportion of hospitals in the high quality category compared with simple additive and PCA-based scoring mechanisms. There was less variation among high scoring facilities according to scoring mechanisms by managing authority and locality except in Malawi. In Malawi, the PCA-based approach assigned a much larger proportion of private and urban facilities to the high quality category compared with the weighted approach. Between the weighted additive and PCA-based scores, the percentage point differences between the proportion of high quality facilities was 34 percentage points among privately run facilities and 40 percentage points among urban facilities.

## Discussion

### Summary of findings

This study calculated summary indices of quality of care using three mechanisms: a simple additive approach, a weighted additive approach, and principal components analysis (PCA). We assessed the amount of agreement between these mechanisms in how they rank facilities by level of quality of care. When comparing the three types of scoring mechanisms, we found that the agreement between the scores in the levels in which facilities were categorized varied both by the method used to compute the index as well as by country. Most often, the lowest agreement occurred between the weighted additive and PCA scores. In both Haiti and Tanzania, the highest agreement was between the simple additive score and the PCA score. This may be explained by the consistently low loadings, which resulted in a lack of variation in PCA-based weights applied to the items, resembling more equally weighted items as in the simple additive score. In Malawi, there was a larger range in the item loadings, including many items with negative loadings. The results of our study are consistent with another study that analyzed the agreement between scoring methods [13]; the authors compared a simple additive approach with a PCA-based approach and identified high correlation between scores. The researchers also noted differences after they categorized the scores into quintiles [13].

Because of the differences in each set of observations or dataset, PCA will not necessarily produce consistent results across different contexts, as seen in our study. Additionally, as our results also show, the variables that load highest on the first component may not align with most important theoretical aspects of the latent construct. In the study by Fruhauf et al [27] that attempted to create quality indices with a number of structural indicators, the PCA resulted in a first component that explained 42% to 79% of variance in four different settings. In such circumstances, it is appropriate to weight indicators based on the loadings from the first component. However, in our study, the first component from PCA explains a low proportion of variance (10% or less), which indicates the quality of care may be multidimensional and using the loadings from only the first component of PCA may also be an arbitrary method for assigning weights.

Researchers have circumvented this issue of a low proportion of variance explained by the first component by using PCA or factor analysis to identify the critical dimensions of a construct and calculate separate “sub-scales”, or separate indices for each dimension, based on the items that most strongly represent that dimension. PCA would only be applied to create a sub-scale only where the proportion of variance explained by the eigenvalue was sufficient [40–44].

These complex considerations may render the use and understanding of a PCA-based score unrealistic for policymakers or lay persons. As evidenced in this paper, when using a PCA approach, scores may not be comparable across datasets; therefore, additive approaches may be particularly salient when comparing across countries or over time for benchmarking purposes. Given the limitations of a simple additive approach, we recommend that policy and program stakeholders should adopt a weighted additive scoring mechanism, especially when summarizing a large number of highly correlated indicators. The WHO uses this approach to create an index of service availability [23].

### Limitations of the study

A key limitation of this analysis was the large number of indicators. This likely contributed to the overall low loadings produced by the PCA. The eigenvalue of each component represents the variance explained by that component, which can be calculated by summing the squared loadings of each item in that component. That is, each item’s loading on that component represents the unique variance it contributes to that particular component’s variance. Therefore, the larger the number items, the more the variance of a component will need to be divided, thus reducing the potential variance each item can contribute to that component. These loadings are further reduced within highly dimensional constructs, where the total variance is spread across multiple components. With as many variables in the analysis, it is unclear whether the inconsistent PCA results, or results incongruent with theory was an artifact of the number of variables or the concept itself. Given fewer variables, perhaps the PCA may have produced results that more strongly aligned with theory, more consistent across the three countries, and perhaps shown different levels of agreement with other scoring mechanisms.

Our study included only family planning facilities that had observation data, or clients available for observation of family planning services on the day of the assessment. There may be important differences between these facilities and facilities without family planning clients present for observation. S3 Table shows the availability of structural quality items among facilities with and without client data. Facilities with client data tend to be better prepared to provide family planning services across all three countries. The higher levels of availability of these items may have introduced bias into each scoring approach, although the extent of impact to each approach is unknown.

### Suggestions for future research

The large number of indicators of quality of care for family planning complicates both data collection processes and calculation of summary measures. After several years of research on these indicators, MEASURE Evaluation (then The Evaluation Project) identified a core list of 25 indicators related to family planning [45]. Researchers continue to evolve these indicators for quality assessment tools [7], although as of 2016, there have been no formal, published validation studies [16]. A validated set of indicators could benefit this field of study.

Further quantitative and qualitative research can expand our understanding of indices of quality of care. PCA a qualitative tool that can be used for data reduction, although our analysis resulted in inconsistent findings across countries. Latent class analysis could d identify the indicators of quality that associate with the latent category of high-quality health facilities. Qualitative methodologies, like the Delphi method, which is based on interviews with subject matter experts, may also produce a reduced set of indicators.

## Conclusions

Simple additive scores are critiqued for being unsophisticated, failing to consider the greater relative importance of some indicators over others. Others adopt more complex scoring mechanism, such as applying weighting schemes based on predetermined weights or from multivariate analyses such as PCA. In our study, the PCA yielded results that suggest that the most critical indicators of quality of care vary across country. The low loadings produced by the PCA created scores most similar to simple additive measures in two of the three countries, resulting in consistency between simple additive scores and PCA-based scores in the way they rank the quality of care at health facilities. Moreover, the PCA results confirm that the construct of quality is multidimensional, the implications being that researchers should consider the use of sub-scales if possible. If a summary index must be used, we suggest a weighted additive summary measure, as it could be more useful and intuitive from the perspective of program planning. Simpler to construct and easier to interpret than a PCA-based summary measure, weighted additive measures can overcome shortcomings of the simple additive measures by accounting for issues of dimensionality and collinearity.

## Supporting information

S1 Table Percent distribution of facilities with family planning services and family planning clients observed on the day of the survey, by facility characteristics. (DOCX)

S2 Table Availability of structure items among facilities with and without client observation data. (DOCX)

S3 Table Percent distribution of facilities by facility background characteristic and level of quality of care. (DOCX)
