# Supplementary material for: Comparing summary measures of quality of care for family planning in Haiti, Malawi, and Tanzania
Source: PLoS One. 2019 Jun 7;14(6):e0217547. doi: 10.1371/journal.pone.0217547 (PMC6555515; doi:10.1371/journal.pone.0217547)
Supplement: S1 Table — (DOCX) [file pone.0217547.s001.docx]

S1 Table. Percent distribution of facilities with family planning services and family planning clients observed on the day of the survey, by facility characteristics

|  | Haiti | Malawi | Tanzania |
| --- | --- | --- | --- |
| **Facility Type** |  |  |  |
| Hospital | 12.8 | 14.8 | 6.5 |
| Primary^1^ | 1.7 | 0.8 | 0.9 |
| Secondary^2^ | 1.7 | 6.2 | 0.6 |
| Tertiary/Other^3^ | 9.4 | 7.8 | 5.2 |
| Health center, dispensary, other | 87.2 | 85.2 | 93.5 |
| Health center/Maternity^4^ | 48.2 | 55.9 | 15.5 |
| Dispensary/Clinic^5^ | 39.0 | 29.3 | 78.0 |
| **Managing Authority** |  |  |  |
| Public | 48.6 | 63.9 | 86.9 |
| Private | 51.4 | 36.1 | 13.1 |
| **Locale** |  |  |  |
| Rural | 59.3 | 70.3 | 71.9 |
| Urban | 40.7 | 29.7 | 28.1 |
|  |  |  |  |
| **Total** | **405** | **371** | **398** |

^1^ Primary hospitals in Haiti include university hospitals, central hospitals in Malawi, and national referral hospitals in Tanzania.

^2^ Secondary hospitals in Haiti include departmental hospitals; district hospitals in Malawi, and regional hospitals in Tanzania.

^3^ Tertiary/other hospitals in Haiti include community reference hospitals, community or rural hospitals in Malawi, and district hospitals in Tanzania.

^4^ Health centers in Haiti include health centers both with and without beds; only Malawi has maternity-designated facilities.

^5^ There are no designated clinics in Haiti; in Malawi, clinics are a lower tier than dispensaries; in Tanzania, clinics are a higher tier than dispensaries although less than one percent of facilities with family planning clients are clinics.
