# Supplementary material for: Comparing summary measures of quality of care for family planning in Haiti, Malawi, and Tanzania
Source: PLoS One. 2019 Jun 7;14(6):e0217547. doi: 10.1371/journal.pone.0217547 (PMC6555515; doi:10.1371/journal.pone.0217547)
Supplement: S2 Table — (DOCX) [file pone.0217547.s002.docx]

S2 Table. Percent distribution of facilities by facility background characteristic and level of quality of care

| **Haiti** |  |  | | | |  | | |  | | | | |  |  | | |  | | |  | | | |  | |  | | |  | | |  | | |  | |
| --- | --- | --- | --- | --- | --- | --- | --- | --- | --- | --- | --- | --- | --- | --- | --- | --- | --- | --- | --- | --- | --- | --- | --- | --- | --- | --- | --- | --- | --- | --- | --- | --- | --- | --- | --- | --- | --- |
|  | **Simple additive** | | | | | | | | | | | | | **Weighted additive** | | | | | | | | | | | **PCA** | | | | | | | | | | **N** | | |
|  | | | Low | | Medium | | | High | | | Total | | Low | | | | Medium | | | High | | | Total | | | Low | | | Medium | High | | Total | | |  |  |  |
| **Facility Type** | | |  | |  | | |  | | |  | |  | | | |  | | |  | | |  | | |  | | |  |  | |  | | |  | | |
| Hospital | | | 11.5 | | 42.3 | | | 46.2 | | | 100.0 | | 19.2 | | | | 30.8 | | | 50.0 | | | 100.0 | | | 15.4 | | | 30.8 | 53.8 | | 100.0 | | | 52 | | |
| Health center,  dispensary, other | | | 40.0 | | 35.7 | | | 24.3 | | | 100.0 | | 35.5 | | | | 33.7 | | | 30.8 | | | 100.0 | | | 36.1 | | | 33.7 | 30.2 | | 100.0 | | | 353 | | |
| **Managing authority** | | |  | |  | | |  | | |  | |  | | | |  | | |  | | |  | | |  | | |  |  | |  | | |  | | |
| Public | | | 38.6 | | 38.6 | | | 22.8 | | | 100.0 | | 37.1 | | | | 32.5 | | | 30.4 | | | 100.0 | | | 34.6 | | | 34.0 | 31.4 | | 100.0 | | | 197 | | |
| Private | | | 34.3 | | 34.6 | | | 31.1 | | | 100.0 | | 29.9 | | | | 34.1 | | | 36.0 | | | 100.0 | | | 32.3 | | | 32.7 | 35.0 | | 100.0 | | | 208 | | |
| **Locale** | | |  | |  | | |  | | |  | |  | | | |  | | |  | | |  | | |  | | |  |  | |  | | |  | | |
| Rural | | | 44.2 | | 37.1 | | | 18.7 | | | 100.0 | | 35.9 | | | | 36.6 | | | 27.5 | | | 100.0 | | | 42.2 | | | 36.2 | 21.6 | | 100.0 | | | 240 | | |
| Urban | | | 24.9 | | 35.8 | | | 39.3 | | | 100.0 | | 29.8 | | | | 28.5 | | | 41.7 | | | 100.0 | | | 20.7 | | | 29.1 | 50.2 | | 100.0 | | | 165 | | |
|  | | |  | |  | | |  | | |  | |  | | | |  | | |  | | |  | | |  | | |  |  | |  | | |  | | |
| **Total** | | | 36.4 | | 36.5 | | | 27.1 | | | 100.0 | | 33.4 | | | | 33.3 | | | 33.3 | | | 100.0 | | | 33.4 | | | 33.3 | 33.2 | | 100.0 | | | 405 | | |
| **Malawi** | | |  |  | | |  | | |  | |  | | | |  | | |  | | |  | |  | | | |  | | |  | | |  | | |  |
|  | | | **Simple additive** | | | | | | | | | | | **Weighted additive** | | | | | | | | | | | **PCA** | | | | | | | | | | **N** | | |
|  | | | Low | | Medium | | | High | | | Total | | Low | | | | Medium | | | High | | | Total | | | Low | | | Medium | High | | Total | | |  |  |  |
| **Facility type** | | |  | |  | | |  | | |  | |  | | | |  | | |  | | |  | | |  | | |  |  | |  | | |  | | |
| Hospital | | | 12.2 | | 24.6 | | | 63.2 | | | 100.0 | | 26.2 | | | | 36.9 | | | 36.8 | | | 100.0 | | | 17.5 | | | 29.9 | 52.6 | | 100.0 | | | 55 | | |
| Health center,  dispensary, other | | | 41.7 | | 31.0 | | | 27.3 | | | 100.0 | | 34.6 | | | | 32.9 | | | 32.5 | | | 100.0 | | | 36.3 | | | 33.9 | 29.9 | | 100.0 | | | 316 | | |
| **Managing authority** | | |  | |  | | |  | | |  | |  | | | |  | | |  | | |  | | |  | | |  |  | |  | | |  | | |
| Public | | | 35.2 | | 32.1 | | | 32.8 | | | 100.0 | | 28.5 | | | | 34.1 | | | 37.5 | | | 100.0 | | | 45.9 | | | 35.6 | 18.5 | | 100.0 | | | 237 | | |
| Private | | | 41.1 | | 26.5 | | | 32.3 | | | 100.0 | | 42.0 | | | | 32.5 | | | 25.5 | | | 100.0 | | | 11.7 | | | 29.2 | 59.1 | | 100.0 | | | 134 | | |
| **Locale** | | |  | |  | | |  | | |  | |  | | | |  | | |  | | |  | | |  | | |  |  | |  | | |  | | |
| Rural | | | 39.0 | | 30.8 | | | 30.2 | | | 100.0 | | 28.4 | | | | 36.1 | | | 35.4 | | | 100.0 | | | 43.2 | | | 38.3 | 18.5 | | 100.0 | | | 261 | | |
| Urban | | | 33.3 | | 28.3 | | | 38.4 | | | 100.0 | | 45.0 | | | | 27.3 | | | 27.7 | | | 100.0 | | | 10.7 | | | 21.5 | 67.8 | | 100.0 | | | 110 | | |
|  | | |  | |  | | |  | | |  | |  | | | |  | | |  | | |  | | |  | | |  |  | |  | | |  | | |
| **Total** | | | 37.3 | | 30.1 | | | 32.6 | | | 100.0 | | 33.4 | | | | 33.5 | | | 33.1 | | | 100.0 | | | 33.5 | | | 33.3 | 33.3 | | 100.0 | | | 371 | | |
| **Tanzania** | | |  |  | | |  | | |  | |  | | | |  | | |  | | |  | |  | | | |  | | |  | | |  | | |  |
|  | | | **Simple additive** | | | | | | | | | | | **Weighted additive** | | | | | | | | | | | **PCA** | | | | | | | | | | **N** | | |
|  | | | Low | | Medium | | | High | | | Total | | Low | | | | Medium | | | High | | | Total | | | Low | | | Medium | High | | Total | | |  |  |  |
| **Facility type** | | |  | |  | | |  | | |  | |  | | | |  | | |  | | |  | | |  | | |  |  | |  | | |  | | |
| Hospital | | | 9.0 | | 25.2 | | | 65.8 | | | 100.0 | | 16.2 | | | | 42.5 | | | 41.3 | | | 100.0 | | | 7.7 | | | 15.4 | 76.9 | | 100.0 | | | 26 | | |
| Health center,  dispensary, other | | | 42.7 | | 28.6 | | | 28.8 | | | 100.0 | | 35.3 | | | | 32.5 | | | 32.3 | | | 100.0 | | | 35.5 | | | 34.3 | 30.2 | | 100.0 | | | 372 | | |
| **Managing authority** | | |  | |  | | |  | | |  | |  | | | |  | | |  | | |  | | |  | | |  |  | |  | | |  | | |
| Public | | | 42.1 | | 28.5 | | | 29.4 | | | 100.0 | | 32.4 | | | | 34.6 | | | 31.7 | | | 100.0 | | | 34.2 | | | 34.6 | 31.4 | | 100.0 | | | 346 | | |
| Private | | | 28.9 | | 27.6 | | | 43.5 | | | 100.0 | | 45.2 | | | | 12.7 | | | 42.1 | | | 100.0 | | | 30.2 | | | 24.3 | 45.5 | | 100.0 | | | 52 | | |
| **Locale** | | |  | |  | | |  | | |  | |  | | | |  | | |  | | |  | | |  | | |  |  | |  | | |  | | |
| Rural | | | 41.3 | | 29.5 | | | 29.2 | | | 100.0 | | 35.6 | | | | 31.6 | | | 34.3 | | | 100.0 | | | 34.8 | | | 32.5 | 32.7 | | 100.0 | | | 286 | | |
| Urban | | | 38.2 | | 25.3 | | | 36.5 | | | 100.0 | | 33.9 | | | | 37.1 | | | 29.0 | | | 100.0 | | | 28.1 | | | 30.8 | 34.7 | | 100.0 | | | 112 | | |
|  | | |  | |  | | |  | | |  | |  | | | |  | | |  | | |  | | |  | | |  |  | |  | | |  | | |
| **Total** | | | 40.4 | | 28.3 | | | 31.2 | | | 100.0 | | 34.0 | | | | 33.1 | | | 32.8 | | | 100.0 | | | 33.7 | | | 33.0 | 33.3 | | 100.0 | | | 398 | | |
