# Supplementary material for: Comparing summary measures of quality of care for family planning in Haiti, Malawi, and Tanzania
Source: PLoS One. 2019 Jun 7;14(6):e0217547. doi: 10.1371/journal.pone.0217547 (PMC6555515; doi:10.1371/journal.pone.0217547)
Supplement: S3 Table — (DOCX) [file pone.0217547.s003.docx]

S3 Table. Availability of structure items among facilities with and without client observation data

|  | **Haiti** | | **Malawi** | | **Tanzania** | |
| --- | --- | --- | --- | --- | --- | --- |
|  | % with clients | % without clients | % with clients | % without clients | % with clients | % without clients |
| Choice of methods |  |  |  |  |  |  |
| Mix of methods provided (long acting,  short acting, and barrier) | 46.3 | 19.6 | 68.4 | 58.4 | 60.4 | 51.0 |
| Family planning integration |  |  |  |  |  |  |
| With ANC services | 98.0 | 94.9 | 75.3 | 63.9 | 99.7 | 97.8 |
| With PNC services | 90.8 | 89.2 | 71.1 | 62.0 | 95.0 | 93.7 |
| With STI services | 97.8 | 98.3 | 99.4 | 94.5 | 99.2 | 97.2 |
| With HCT services | 56.8 | 48.4 | 90.3 | 79.0 | 96.5 | 96.6 |
| With PMTCT services | 44.9 | 28.2 | 71.0 | 56.5 | 95.8 | 91.8 |
| Management |  |  |  |  |  |  |
| System for reviewing management/  administrative issues | 60.8 | 51.9 | 60.1 | 53.0 | 72.2 | 72.1 |
| System to obtain client opinions | 3.4 | 1.1 | 9.6 | 4.8 | 15.1 | 8.0 |
| Supervision in the last 6 months | 92.4 | 86.0 | 84.3 | 79.3 | 98.6 | 98.1 |
| Inventory of contraceptive supplies | 57.5 | 39.9 | 86.2 | 74.9 | 66.7 | 66.4 |
| Stock organized by expiration date | 4.2 | 2.6 | 1.6 | 3.4 | 2.5 | 0.6 |
| Contraceptives protected | 66.9 | 56.7 | 76.5 | 72.4 | 55.3 | 57.7 |
| Facility infrastructure |  |  |  |  |  |  |
| *General* |  |  |  |  |  |  |
| Electricity | 66.4 | 59.5 | 61.1 | 56.4 | 66.0 | 60.8 |
| Water | 81.7 | 74.9 | 95.7 | 94.1 | 69.7 | 65.8 |
| Toilet | 42.0 | 44.9 | 32.9 | 34.2 | 38.7 | 32.8 |
| Telephone | 23.2 | 21.0 | 33.9 | 26.0 | 4.9 | 3.7 |
| Waiting area (protected) | 96.3 | 94.9 | 98.2 | 97.4 | 93.2 | 95.6 |
| Quality assurance measures in place | 7.9 | 6.6 | 13.5 | 12.0 | 18.7 | 13.0 |
| *Family planning area* |  |  |  |  |  |  |
| Family planning services provided  5 days per week | 94.3 | 88.6 | 71.7 | 54.2 | 93.0 | 92.1 |
| Private exam room | 94.6 | 90.6 | 97.3 | 95.3 | 93.2 | 94.5 |
| Blood pressure cuff | 90.1 | 88.1 | 69.6 | 70.4 | 79.4 | 69.5 |
| Speculum | 3.4 | 1.7 | 21.1 | 9.0 | 27.9 | 20.1 |
| Family planning guidelines | 61.7 | 37.0 | 38.4 | 34.6 | 64.8 | 52.7 |
| Table and stool | 69.1 | 70.9 | 88.0 | 85.8 | 91.2 | 84.2 |
| Light | 16.0 | 22.5 | 29.2 | 26.5 | 14.2 | 11.8 |
| Soap | 72.3 | 71.8 | 58.4 | 54.9 | 66.7 | 64.4 |
| Gloves | 58.2 | 56.4 | 92.0 | 89.3 | 63.0 | 51.7 |
| Decontamination solution | 65.7 | 58.1 | 58.2 | 56.8 | 59.9 | 55.0 |
| Sharps box | 91.9 | 79.5 | 92.0 | 88.9 | 96.4 | 97.2 |
|  |  |  |  |  |  |  |
| **Number of facilities** | **405** | **351** | **371** | **437** | **398** | **550** |
